# Irisin, Brain-Derived Neurotrophic Factor (BDNF), and Redox Balance in Geriatric Dynapenia

**DOI:** 10.3390/antiox14101268

**Published:** 2025-10-21

**Authors:** Aleksandra Wojszel, Jakub Śliwowski, Justyna Rentflejsz, Joanna Rogalska, Małgorzata Michalina Brzóska, Zyta Beata Wojszel

**Affiliations:** 1 Interdisciplinary Scientific Group at the Department of Geriatrics, Medical University of Bialystok, 15-089 Bialystok, Poland; 42216@student.umb.edu.pl; 2 Doctoral School, Medical University of Bialystok, 15-089 Bialystok, Poland; justyna.rentflejsz@sd.umb.edu.pl; 3 Department of Toxicology, Medical University of Bialystok, 15-089 Bialystok, Poland; joanna.rogalska@umb.edu.pl (J.R.); malgorzata.brzoska@umb.edu.pl (M.M.B.); 4 Department of Geriatrics, Medical University of Bialystok, 15-089 Bialystok, Poland

**Keywords:** sarcopenia, myokine, irisin, neurotrophin, brain-derived neurotrophic factor, total oxidative status, total antioxidative status, oxidative stress index

## Abstract

Irisin and brain-derived neurotrophic factor (BDNF) are considered potential biomarkers for sarcopenia; however, their interplay and relationship with oxidative stress remain unclear. Therefore, the aim of this study was to assess the serum concentration of irisin and BDNF in patients over 60 years of age, as well as their relationship with dynapenia and redox homeostasis. Dynapenia was diagnosed using the Five Times Sit-to-Stand Test (5TSST). Serum levels of irisin, BDNF, total oxidative status (TOS), and total antioxidative status (TAS) were measured, and the oxidative stress index (OSI) was calculated. A total of 110 patients from a geriatric ward (72.7% women, mean age 78.2 ± 7.1 years) participated in the study. BDNF concentration was negatively associated with dynapenia, irisin, and the irisin/BDNF ratio. TOS, TAS, and OSI were negatively associated with BDNF and positively associated with irisin and dynapenia. No significant association was found between irisin and sarcopenia parameters. In regression analysis, significantly higher odds of dynapenia were observed for older age, female sex, a greater number of chronic diseases, and higher OSI values, after adjusting for TOS, BDNF, and the irisin-to-BDNF ratio. These results confirm redox imbalance as an independent predictor of sarcopenia. A lower BDNF concentration and a higher irisin-to-BDNF ratio may indicate a protective role of BDNF in the development of sarcopenia in geriatric patients; however, this finding requires further confirmation.

## 1. Introduction

Sarcopenia, initially considered a geriatric syndrome and a complication of the aging process, was included in the International Classification of Diseases (ICD-10; M62.84) in 2019 and is now recognized as a medical condition that requires diagnosis and treatment. It is primarily characterized by a progressive decrease in skeletal muscle mass and function. Sarcopenia diagnosis is based on anatomical and functional criteria [1]. The latest consensus on sarcopenia prioritized muscle strength as a key prognostic factor and proposed dynapenia as its primary screening criterion. For this purpose, the measurement of grip strength can be used, or the chair stand test result can be employed [2].

Several complex pathogenetic mechanisms are thought to be involved in sarcopenia development, including disturbances in protein homeostasis with a predominance of catabolic over anabolic processes, chronic inflammation, and hormonal imbalances [3]. Therefore, apart from the anatomical biomarkers of sarcopenia proposed in its clinical diagnostic algorithms [1,4,5], several specific biochemical biomarkers have been identified for use in research and clinical trials. They can be specified as markers of musculoskeletal status (myokines, markers of the neuromuscular junction, those specific to muscle mass and muscle turnover) and markers of processes underlying the pathogenesis of sarcopenia (adipokines, hormones, and inflammatory parameters) [6]. Among them, irisin and brain-derived neurotrophic factor (BDNF) are two substances regarded as potential biomarkers for sarcopenia. Irisin is a myokine, a polypeptide compound secreted by muscles in response to physical exercise. It can prevent muscle degradation associated with aging through its antioxidant properties and influence the regeneration and growth of muscle tissue by activating the appropriate signaling pathways [7]. BDNF is a neurotrophin that may play a crucial role in regulating neuromuscular junction function, which is essential not only for nerve–muscle coordination but also for maintaining muscle strength and function [8]. Studies conducted in recent years indicate a link between the secretion of irisin during physical exercise and the expression of BDNF (named the muscle–brain axis) [9]. However, the mutual interaction of these substances in the development of sarcopenia is unclear. Especially in the context of oxidative stress, an essential player in the complex pathomechanisms of sarcopenia development [10].

Therefore, the study aimed to measure the serum concentration of irisin and BDNF in older geriatric patients and to assess their association with markers of dynapenia and redox homeostasis parameters.

## 2. Materials and Methods

The study was conducted with patients over 60 years of age in the sub-acute geriatric ward who were able to participate in the functional assessment. The study was approved by the Bioethics Committee at the Medical University of Bialystok (no.APK.002.513.2022). All procedures performed in the study were conducted following the ethical standards of the Medical University of Bialystok’s research committee and in compliance with the Helsinki Declaration and its subsequent amendments. All study participants provided their informed consent to participate. Patients participated in the study voluntarily, knowing they could withdraw at any time, and the study was not burdensome for them. Functional assessment was part of a routine comprehensive geriatric assessment at the department, and blood samples for additional biochemical tests were collected during scheduled blood draws. Serum samples were obtained from fasting venous blood and then stored at −80 °C until assayed.

The inclusion criteria were: age 60 years or older, provision of informed consent, stable clinical condition, and health and fitness status sufficient to allow participation in a functional assessment.

Exclusion criteria included: neuromuscular disorders such as Parkinson’s disease and amyotrophic lateral sclerosis; moderate to severe dementia (defined as a Mini-Mental State Examination score of <18 points); advanced or terminal stages of chronic diseases (e.g., severe chronic obstructive pulmonary disease requiring oxygen therapy, severe cardiovascular disease, decompensated heart failure, and severe osteoarthritis); active phases of acute illnesses; the presence of a pacemaker; ongoing cancer treatment; and lack of informed consent.

Information on patient’s age, gender, and number of chronic diseases frequently occurring in geriatric patients, associated with the risk of sarcopenia, and in the pathogenesis of which oxidative stress plays an important role (out of 19: atrial fibrillation, chronic arthritis, chronic cardiac failure, chronic obstructive pulmonary disease, asthma, chronic renal disease, dementia, depression, urinary incontinence, diabetes/prediabetes, hypertension, orthostatic hypotension, ischemic heart disease, myocardial infarction, neoplasm, osteoporosis, peripheral arterial disease, and stroke) was collected; two or more diseases was classified as “multimorbidity”.

Screening for sarcopenia included:

- The SARC-F questionnaire (an acronym that stands for Strength, Assistance in walking, Rise from a chair, Climb stairs, and Falls), a self-assessment tool for identifying deficiencies suggestive of sarcopenia. A score of 0 to 2 points may be assigned for each item (with a maximum total score of 10). A result of ≥4 points indicates probable sarcopenia and a potential risk of poor functional outcomes [11,12].
- Functional test assessing the strength of lower extremities—5 Times Sit-to-Stand Test, 5TSST. The patient sits in a chair with a straight back, feet flat on the floor, and arms folded across their chest. The time it takes the person to stand up and sit down five times as quickly as possible is measured. A result greater than 15 seconds indicates lower limb dynapenia, which is recognized as probable sarcopenia.

The serum concentration of irisin was determined with the use of a Human Irisin ELISA Kit (No. EH4702) and that of BDNF with the use of a Human BDNF ELISA Kit (No. EH0043) by FineTest (Wuhan, China). The kits were based on sandwich enzyme-linked immunosorbent assay technology (ELISA). The precision of the irisin assay, expressed as intra- and inter-assay coefficients of variation (CV), was <2% (for both used kits, intra-assay CV <2%) and 2%, respectively. The intra-assay CV of the BDNF measurement was <2% for the first kit and <4% for the second kit, while the inter-assay CV was <3%. The ratio of irisin to BDNF (irisin/BDNF) was mathematically calculated.

Total oxidative status (TOS) and total antioxidative status (TAS) were measured in the serum using spectrophotometric PerOx (TOS/TOC) Kit (No. KC5100) and ImAnOx (TAS/TAC) Kit (No. KC5200), respectively, provided by Immundiagnostik AG (Bensheim, Germany). To assay TOS, the total lipid peroxides present in the serum were quantified through the reaction of peroxidase and peroxides in the tested sample, as well as the transformation of 3,3′,5,5′-tetramethylbenzidine added to the sample into a colored product. TAS was determined based on the reaction of antioxidants present in the serum and the added hydrogen peroxide. TOS values measured in control samples included with the first kit (271.0 ± 11.31 µmol/L and 602.5 ± 13.44 µmol/L; mean ± standard error for two repetitions) and the second kit (141.8 ± 3.111 µmol/L and 352.0 ± 6.364 µmol/L) fell within the ranges specified by the manufacturer (first kit 204–338 µmol/L and 450–750 µmol/L, respectively; second kit 122–203 µmol/L and 260–433 µmol/L, respectively). The intra-assay CV was <2% for the first kit and <4% for the second kit. TAS values determined in control samples included with the first kit (221.58 ± 6.611 µmol/L and 265.0 ± 18.38 µmol/L; mean ± standard error for two repetitions) and the second kit (214.66 ± 5.037 µmol/L and 252,1 ± 5374 µmol/L) fell within the ranges specified by the manufacturer (180–242 µmol/L and 212–286 µmol/L in both kits). The intra-assay CV was <1% for the first kit and <2% for the second kit, while the inter-assay CV was <2%. Serum assays were performed in duplicate to ensure the reliability, precision, and validity of the results, and to enhance the diagnostic and analytical value of the obtained data, using an Epoch spectrophotometer (BioTek Instruments, Inc., Winooski, VT, USA).The oxidative stress index (OSI) was mathematically calculated as the ratio of TOS and TAS (OSI = TOS/TAS) [13].

Statistical analysis was performed using IBM SPSS Version 18 software (SPSS, Chicago, IL, USA). The distribution of variables was checked using the Shapiro–Wilk test and presented as frequencies and percentages (categorical variables), as means and standard deviations (normally distributed quantitative variables), or as medians and interquartile ranges (non-normally distributed quantitative variables). Proportions were compared using χ^2^ tests, while Student’s *t*-test for independent samples and the Mann–Whitney U test were used to compare means and medians. Spearman’s correlation was used to assess the relationship between variables. Multivariable logistic regression models of dynapenia, as the dependent variable, were built, including all predictors with a *p*-value less than 0.1, excluding those with a high multicollinearity effect. In all analyses, a two-tailed *p*-value of less than 0.05 was regarded as significant.

## 3. Results

### 3.1. Study Group Characteristics

One hundred ten patients from the geriatric ward (72.7% women, mean age 78.2 ± 7.1 years) participated in the study. Table 1 presents the characteristics of the study group. Dynapenia of the lower extremities was more frequently observed in older patients and in females.

Dynapenia, assessed with the 5-TSST, occurred in as many as 66.4% of the study group, significantly more often in those with SARC-F scores of 4 or higher. Individuals with reduced lower limb muscle strength had a considerably higher number of chronic diseases and were substantially more frequently diagnosed with multimorbidity.

### 3.2. Association Between Dynapenia, Irisin, BDNF and Redox Parameters

The analysis revealed that BDNF concentration was significantly lower in patients with dynapenia, while there was no significant association between dynapenia and irisin. The irisin/BDNF ratio was considerably higher in dynapenic patients. TAS did not differ significantly between the two analyzed groups. In contrast, TOS was higher in the dynapenia group (*p* on the verge of significance, 0.05), and OSI was significantly higher in the dynapenia group (Table 2).

5TSST results were positively associated with age, the number of chronic diseases, SARC-F scores, the irisin/BDNF ratio, TAS, and OSI and negatively associated with BDNF concentration (Table 3). BDNF concentration was negatively associated with irisin concentration, the irisin/BDNF ratio, TOS, TAS, and OSI. In contrast, irisin concentration was positively associated with the irisin/BDNF ratio, TOS, TAS, and OSI.

The concentrations of irisin and BDNF, as well as their ratio, were not associated with age or the number of chronic diseases. TAS showed a negative association with the number of chronic diseases.

### 3.3. Predictors of Dynapenia in Regression Analysis

A direct multivariable logistic regression analysis was conducted to investigate dynapenia as the outcome and seven predictors: age, sex, number of chronic diseases, OSI, TOS, BDNF concentration, and the irisin/BDNF ratio (Table 4). Significantly higher odds for dynapenia were observed only for female sex (odds ratio, 5.45; 95% CI, 1.75–16.96; *p* = 0.003), age (odds ratio, 1.13; 95% CI, 1.04–1.23; *p* = 0.005), and number of chronic diseases (odds ratio, 1.48; 95% CI, 1.01–2.16; *p* = 0.04). For the model, an overall prediction success rate of 81.8% was observed, with 93.2% of the dynapenia “+” status (sensitivity) and 59.5% of dynapenia “−“ status (specificity) correctly predicted.

A stepwise backward logistic regression analysis was performed on dynapenia, using seven predictors to construct Model 1 (Table 5). The results of the study suggested the model with four variables only: age (odds ratio, 1.13; 95% CI, 1.04–1.22; *p* = 0.003), female sex (odds ratio, 5.67; 95% CI, 1.86–317.27; *p* = 0.002), number of chronic diseases (odds ratio, 1.44; 95% CI, 1.01–2.06; *p* = 0.04), and OSI (odds ratio, 2.20; 95% CI, 1.23–3.94; *p* = 0.008). Prediction success was slightly worse in the case of Model 2, with 90.4% of dynapenia “+” cases and 79.1% of dynapenia “−” cases correctly predicted, resulting in an overall success rate of 79.1%.

## 4. Discussion

The study has confirmed a high prevalence (66.9%) of dynapenia, as assessed with the 5TSST, among geriatric patients. It was positively associated with older age, female sex, and the number of chronic diseases. Additionally, the time required to perform the test increased with age and the number of diseases diagnosed. This finding is consistent with previous research results, which confirm that muscle strength declines with age and is also associated with several diseases [14,15]. All expert groups involved in the development of existing algorithms for sarcopenia diagnosis emphasize the crucial role of muscle strength in assessment. In older adults, it is a predictor of adverse health-related outcomes (such as mobility limitation, falls, activities of daily living disability, and mortality in community-dwelling older adults) [5].

In our study, the 5TSST results and the diagnosis of dynapenia were not associated with irisin concentration; however, they showed a positive relationship with the irisin-to-BDNF ratio and a negative relationship with BDNF concentration. According to the latest systematic review on the relationship between irisin and sarcopenia, cross-sectional studies have demonstrated lower serum irisin concentration in individuals with sarcopenia compared to healthy controls [16,17]. The majority of the available 12 human studies results suggested that irisin might serve as a potential diagnostic marker for sarcopenia in the older population and postmenopausal women. Moreover, animal and cellular experiments indicated that increased concentrations of irisin helped improve muscle mass [7]. However, the authors of this review noted that the majority of the studies analyzed had a high or unclear risk of selection bias, performance bias, and detection bias. They also pointed out that conflicting evidence exists, as some researchers failed to establish a definitive connection between irisin and sarcopenia [7,18]. Our study also did not confirm that the concentration of irisin itself differed significantly between patients with and without dynapenia, suggesting that irisin may not accurately predict sarcopenia in older, multimorbid individuals. In a recent study conducted among sedentary adults, a decrease in irisin concentrations was observed after an 8-week intensive lifestyle intervention program [19]. This finding was contrary to its supposed mechanisms of action and dynamics, suggesting that there may be yet undiscovered impacts on the secretion of irisin [19]. A recently published analyses of research on irisin in the context of its association with age-related problems, including sarcopenia, showed that the evidence is not consistent regarding the association between irisin concentration and health or disease in older adults and indicated the need for further research in this area, taking into account the influence of various factors, including not only age, lifestyle, diet, and ethnicity, but also comorbidities and concomitant medications [20].

BDNF is a neurotrophin produced primarily by the brain, involved in synaptic modulation and neurogenesis, influencing neuronal survival and brain angiogenesis [21,22,23]. It has also been shown to be a myokine, a molecule synthesized in skeletal muscle that controls biological processes occurring within it. BDNF is believed to play a role in cellular mechanisms that regulate muscle function, maintenance, and plasticity [8]. When released during exercise, BDNF directly contributes to the strengthening of neuromuscular junctions [24] as well as insulin-regulated glucose uptake and beta-oxidation processes in muscle tissue. It is primarily produced in contracting skeletal muscle and plays a vital role in metabolic regeneration and exercise-induced skeletal muscle remodeling [8,25]. Some studies have confirmed that its concentration in the blood significantly increases during physical activity [26,27]. In contrast, a meta-analysis of studies on the relationship between BDNF in the blood and physical activity in patients with diabetes showed that the results obtained by different researchers were inconsistent—some confirmed that BDNF level increases, others that it decreases, and in some studies, no significant effect of exercise on BDNF concentration was observed [28]. Perhaps the type of physical activity undertaken by the respondents may also have an impact; moderate-intensity exercises seem to be more effective in promoting an increase in the peripheral levels of BDNF in older adults [29]. Nevertheless, extraocular eye muscles are those that are less susceptible to age-related loss of function and mass. It has been confirmed that the satellite cells of these muscles express higher levels of neurotrophins, including BDNF, and their receptors compared to limb muscles [30]. Some researchers suggested that neurotrophins may be hidden behind their highly regenerative properties and play a role in protecting craniofacial muscles against inflammatory and wasting diseases [31].

Some studies suggest that irisin, secreted during physical exercise, can cross the blood–brain barrier and enhance BDNF expression [32,33,34]. However, in our study, irisin concentration showed the inverse relationship with BDNF, further highlighting the complexity of the interaction between these two molecules. Our study demonstrated that the time required to perform the 5TSST and the occurrence of dynapenia were inversely associated with BDNF concentration and positively associated with the irisin-to-BDNF ratio. These findings may support the protective role of this neurotrophin in the development of sarcopenia in geriatric, multimorbid patients, and highlight the importance of the irisin-to-BDNF ratio in the context of sarcopenia.

Oxidative stress and mitochondrial dysfunction have been considered as critical pathophysiologic mechanisms involved in the development of sarcopenia [10,35,36]. Skeletal muscle is particularly susceptible to oxidative stress-related damage and dysfunction because it is an organ characterized by a high metabolic rate [37,38]. TOS, TAS, and OSI are parameters of redox homeostasis. TOS evaluates the overall oxidative state of the body. At the same time, TAS measures the overall antioxidative status, while OSI reflects the imbalance between the oxidative-reductive processes, considered a most precise indicator of oxidative stress in the body [13,39]. The results of our study indicate that TOS and OSI were strongly associated with the occurrence of dynapenia in patients in the geriatric ward. A positive association was found between these redox homeostasis markers and both the longer time needed to perform the 5TSST and the frequency of dynapenia diagnosis based on this test. No association was found with TAS. It suggests that antioxidative mechanisms are not sufficiently effective, and the redox balance has shifted towards oxidation in the studied group of people with dynapenia. The regression analysis has confirmed that OSI was the only significant biochemical marker of sarcopenia in the studied group of geriatric patients, controlling for age, sex, and the number of chronic diseases. Several studies have confirmed that oxidative stress may contribute to sarcopenia by affecting the neuromuscular junction, leading to loss of innervation, a negative impact on muscle mitochondrial function, and promoting atrophy pathways, ultimately disrupting muscle contractile function [10].

This study has several limitations that warrant emphasis. First, as noted above, the term “dynapenia” used in the manuscript refers specifically to reduced lower extremity muscle strength, as assessed by the Five Times Sit-to-Stand Test (5TSTS). Some studies suggest that lower limb muscle weakness, in particular, is a strong predictor of adverse outcomes in older adults [40]. However, the evidence is not entirely consistent, and handgrip strength (HGS) is often cited as a more reliable indicator of overall muscle strength and a key diagnostic criterion for sarcopenia [41,42]. That said, HGS measurement also has its limitations, as highlighted by experts. These include patient-related factors common in older populations—such as pain due to osteoarthritis, fatigue, or low motivation (e.g., in patients with depression)—as well as measurement-related factors, including variability in testing protocols and differences in dynamometer design and calibration [43,44,45]. Nevertheless, at the time this study was conducted, the prevailing consensus on sarcopenia diagnosis recognized the Five Times Sit-to-Stand Test as a valid alternative for assessing muscle strength when HGS measurement was not feasible [2].

The 5TSST test is also a fitness test, and we cannot rule out the possibility that it may not accurately reflect general muscle strength, mass, and quality, providing a reasonable basis for research on biochemical markers of dynapenia/sarcopenia. Additionally, the first global consensus on the conceptual definition of sarcopenia, published in 2024, recommended that muscle mass, muscle strength, and muscle-specific force be included in the diagnostic algorithm, while physical performance is viewed as an outcome [1].

The results of this study can be generalized to populations with similar characteristics—namely, individuals of more advanced age and with a high burden of multimorbidity—but not to the broader population of older adults. It is possible that the specific characteristics of this group, being relatively older and with a higher prevalence of disease compared to cohorts previously studied for BDNF or irisin levels, also influenced the results obtained, including the finding that redox imbalance was a significant, independent predictor of dynapenia.

The results of our study suggest a potential protective role of BDNF; however, longitudinal studies and more specific measures of neuromuscular function are needed to confirm this directly. Additionally, although the irisin/BDNF ratio appears to be a promising indicator of sarcopenia, its interpretation remains uncertain—it is unclear whether it represents a reliable biomarker or merely reflects a state of physiological imbalance. As this was an observational study, we cannot definitively determine the direction of causality between OSI and reduced muscle strength. Moreover, several confounding factors that influence oxidative stress—such as diet, physical activity, inflammatory status, or medication use—were not taken into account. These limitations should be addressed in future research.

Furthermore, our study was static and lacked any intervention involving physical activity. In the available literature, these myokines have often been assessed using dynamic models, particularly in the context of the impact of physical exercise on their concentrations [9]. Additionally, several studies have confirmed that irisin levels are influenced not only by physical activity but also by metabolic health conditions such as obesity and insulin resistance. Its concentration in the body is associated with the amount of intramuscular fat tissue, which is actively utilized during exercise [46,47,48,49,50]. It is possible that different mechanisms are responsible for baseline levels and exercise-induced changes in these myokines.

## 5. Conclusions

The study results have confirmed a lower status of BDNF and a higher irisin/BDNF ratio in patients with dynapenia, suggesting that lower BDNF expression may occur in aging muscles despite adequate irisin concentration. Higher levels of oxidative stress parameters can also play a role in the development of sarcopenia.

A lower concentration of BDNF and a higher irisin/BDNF ratio in patients with dynapenia may indicate a protective role of BDNF in the development of sarcopenia in geriatric patients. Higher levels of oxidative stress parameters point to redox imbalance involvement in sarcopenia development. Older age, female gender, and the higher number of chronic diseases and OSI were the only independent predictors connected with significantly higher odds for dynapenia status, controlling for age, sex, and number of chronic diseases. The findings may suggest the need to enhance the body’s antioxidant potential to reduce the severity and frequency of dynapenia in older patients.

## Figures and Tables

**Table 1:** Study group characteristics.

Characteristic	Total	Dynapenia “−”	Dynapenia “+”	*p* ^1^
N (%)	110 (100)	37 (33.6)	73 (66.4)	
Age (y), M (SD)	78.2 (7.1)	74.3 (7.8)	80.1 (5.9)	<0.001
Sex				0.003
female, n (%)	80 (72.7)	20 (25.0)	60 (75.0)	
male, n (%)	30 (27.3)	17 (56.7)	13 (43.3)	
SARC-F, points, Me (IQR)	2.0 (1.0, 4.0)	1 (0, 2)	3 (1.5, 5)	<0.001
Sarcopenia by SARC-F				<0.001
no (<4 points), n (%)	74 (67.3)	36 (48.6)	38 (51.4)	
yes (≥4 points), n (%)	36 (32.7)	1 (2.8)	35 (97.2)	
Number of chronic diseases, Me (IQR)	4 (3, 5)	3 (2, 4.5)	4 (3, 6)	0.003
Multimorbidity				0.006
no, n (%)	7 (6.4)	6 (85.7)	1 (14.3)	
yes, n (%)	103 (93.6)	31 (30.1)	72 (69.9)	
5TSST, s, Me (IQR)	17.4 (13.5, 24.0)	12.9 (11.7, 13.6)	20.4 (17.4, 28.0)	<0.001

IQR—Interquartile Range; M—Mean; Me—Median; N—number; SD—Standard Deviation; 5TSST—5 Times Sit-to-Stand Test. ^1^ Student’s *t*-test or the Mann–Whitney U test. Multimorbidity—2 or more diseases of 19 assessed. Dynapenia “−”—5TSST ≤ 15 s; Dynapenia “+”—5TSST > 15 s.

**Table 2:** Dynapenia and irisin, BDNF, irisin/BDNF ratio and redox balance parameters.

Characteristic	All	Dynapenia “−”	Dynapenia “+”	*p* ^1^
N (%)	110 (100)	37 (33.6)	73 (66.4)	
BDNF, ng/mL, Me (IQR)	4.02 (2.4, 5.9)	5.45 (3.1, 6.9)	3.7 (2.9, 5.6)	0.03
Irisin, µg/mL, Me (IQR)	2.1 (1.7, 2.4)	1.9 (1.6, 2.3)	2.2 (1.7, 2.4)	0.11
Irisin/BDNF ratio, Me(IQR)	584 (272, 760)	379 (230, 705)	648 (297, 773)	0.02
TOS, µmol/L, Me (IQR)	656.5 (401.1, 1041.4)	495.4 (399.7, 818.1)	799.6 (401.9, 1111.1)	0.05
TAS, µmol/L, Me (IQR)	359.4 (277.4, 386.1)	375.8 (265.4, 386.6)	353.1 (291.6, 385.1)	0.94
OSI, M (SD)	2.1 (1.4, 2.8)	1.9 (0.7)	2.3 (1.1)	0.03

BDNF—brain-derived neurotrophic factor; IQR—Interquartile Range; M—Mean; Me—Median; N—number; OSI—oxidative stress index; SD—Standard Deviation; TAS—total antioxidative status; TOS—total oxidative status; ^1^ Student’s *t*-test or the Mann–Whitney U test. Dynapenia “−”—5TSST ≤ 15 s; Dynapenia “+”—5TSST > 15 s.

**Table 3:** Spearman’s rank correlation coefficients between the analyzed parameters.

Variable		SARCF	5TSST	BDNF	Irisin	Irisin/ BDNF	TOS	TAS	OSI	Age
SARC-F			0.62 **	−0.11	−0.01	0.06	0.03	−0.09	0.09	0.36 **
	*p*		<0.001	0.24	0.94	0.51	0.78	0.34	0.34	<0.001
5TSST		0.62 **		−0.27 **	0.17	0.27 **	0.27 **	0.06	0.30 **	0.40 **
		<0.001		0.004	0.08	0.005	0.005	0.52	0.001	<0.001
BDNF		−0.11	−0.27 **		−0.53 **	−0.95 **	−0.95 **	−0.95 **	−0.12	−0.12
	*p*	0.24	0.004		<0.001	<0.001	<0.001	<0.001	0.22	0.22
Irisin		−0.01	0.17	−0.53 **		0.71 **	0.55 **	0.69 **	0.33 **	0.04
	*p*	0.94	0.08	<0.001		<0.001	<0.001	<0.001	<0.001	0.71
Irisin/BDNF		0.064	0.27 **	−0.95 **	0.71 **		0.50 **	0.59 **	0.32 **	0.12
	*p*	0.51	0.005	<0.001	<0.001		<0.001	<0.001	<0.001	0.22
TOS		0.03	0.27 **	−0.95 **	0.55 **	0.50 **		0.48 **	0.92 **	−0.07
	*p*	0.78	0.005	<0.001	<0.001	<0.001		<0.001	<0.001	0.47
TAS		−0.09	0.06	−0.95 **	0.69 **	0.59 **	0.48 **		0.18	0.001
	*p*	0.34	0.52	<0.001	<0.001	<0.001	<0.001		0.05	0.99
OSI		0.09	0.30 **	−0.12	0.33 **	0.32 **	0.92 **	0.18		−0.07
	*p*	0.34	0.001	0.22	<0.001	<0.001	<0.001	0.05		0.50
Age (years)		0.36 **	0.40 **	−0.12	0.04	0.12	−0.07	0.001	−0.07	
	*p*	<0.001	<0.001	0.22	0.71	0.22	0.47	0.99	0.50	
Number of diseases		0.38 **	0.33 **	−0.02	−0.03	0.03	−0.10	−0.260 *	−0.04	0.33 **
	*p*	<0.001	<0.001	0.87	0.75	0.76	0.28	0.006	0.69	<0.001

BDNF—Brain Derived Neurotrophic Factor; OSI—Oxidative Stress Index; TOS—Total Oxidant Status; TAS—Total Antioxidant Status; 5TSST—time achieved in 5 Times Sit-to-Stand Test.

**Table 4:** Determinants of dynapenia “+” status—direct multivariable logistic regression model.

	OR	95% CI	*p*
		MODEL 1	
Age (years)	1.13	1.04–1.23	0.005
Sex (female)	5.45	1.75–16.96	0.003
Number of chronic diseases	1.48	1.01–2.16	0.04
OSI	2.23	0.52–9.54	0.28
TOS	1.00	0.996–1.004	0.99
BDNF	0.81	0.61–1.06	0.13
Irisin/BDNF	0.999	0.997–1.001	0.52

BDNF—Brain Derived Neurotrophic Factor; CI—confidence interval; OSI—Oxidative Stress Index; OR—Odds ratio; TOS—Total Oxidative Status.

**Table 5:** Determinants of dynapenia ”+” status—stepwise backward multivariable logistic regression analysis.

	OR	95% CI	*p*
		MODEL 2	
Age (years)	1.13	1.04–1.22	0.003
Sex (female)	5.67	1.86–17.27	0.002
Number of chronic diseases	1.44	1.01–2.06	0.04
OSI	2.20	1.23–3.94	0.008

Variables included in the analysis: age, sex, number of chronic diseases, OSI, BDNF, irisin/BDNF ratio, and TOS. CI—confidence interval; OSI—Oxidative Stress Index; OR—Odds ratio.

## Data Availability

The data that support the findings of this study are available from the corresponding author upon reasonable request. The raw data supporting the findings of this study are available from the corresponding authors upon reasonable request.

## Abbreviations

The following abbreviations are used in this manuscript:

BDNF	Brain-derived neurotrophic factor
CI	Confidence interval
CV	Coefficient of variation
EWGSOP2	The Second European Working Group on Sarcopenia in Older People
ICD-10	The 10th International Classification of Diseases
IQR	Interquartile range
M	Mean
Me	Median
N	Number
OR	Odds ratio
OSI	Oxidative stress index
SARC-F	S(trength), A(ssistance with walking), R(ise from a chair), C(limbing stairs), and F(alls) questionnaire
SD	Standard deviation
TAS	Total antioxidative status
TOS	Total oxidative status
5TSST	5 Times Sit-to-Stand Test
